# Effect of Salt Stress and Foliar Application of Salicylic Acid on Morphological, Biochemical, Anatomical, and Productivity Characteristics of Cowpea (*Vigna unguiculata* L.) Plants

**DOI:** 10.3390/plants11010115

**Published:** 2021-12-31

**Authors:** Ahmed M. El-Taher, Hany S. Abd El-Raouf, Nahid A. Osman, Samah N. Azoz, Magdy A. Omar, Amr Elkelish, Mahmoud A. M. Abd El-Hady

**Affiliations:** 1 Department of Agriculture Botany, Agriculture Faculty, Al-Azhar University, Cairo 11651, Egypt; eltaher69@azhar.edu.eg (A.M.E.-T.); dr-hany70@hotmail.com (H.S.A.E.-R.); MagdyOmar673.el@azhar.edu.eg (M.A.O.); 2 Department of Biology, University College, Taif University, Taif 21944, Saudi Arabia; 3 Department of Science and Technology, Ranya Collage, Taif University, Taif 21944, Saudi Arabia; dnahidosman@hotmail.com; 4 Agricultural Botany, Faculty of Agriculture, Cairo University, Giza 12613, Egypt; 5 Botany Department, Faculty of Science, Suez Canal University, Ismailia 41522, Egypt; amr.elkelish@science.suez.edu.eg; 6 Vegetables and Floriculture Department, Faculty of Agriculture, Damietta University, Damietta 34517, Egypt; m_abdelhady@du.edu.eg

**Keywords:** *Vigna unguiculata* L., salinity stress, SA, growth, productivity, photosynthetic pigments, anatomy

## Abstract

The present study aimed to investigate the impact of salinity on vegetative growth, chemical constituents, and yields of cowpeas (*Vigna unguiculata*) and the possible benefits of salicylic acid (SA) on these plants after damage from salinity. To achieve these objectives, two pot experiments were carried out at the Faculty of Agriculture, Al-Azhar University, Egypt, during the two growing seasons of 2019 and 2020. The results revealed that salinity significantly decreased, and SA treatment substantially increased the plant height, number of compound leaves, number of internodes per plant, fresh weights of leaves and stems, productivity, photosynthetic pigments content, and concentrations of nitrogen (N), phosphorus (P), and potassium (K) of the cowpea plants compared with the control. The anatomical structure of stems and leaves of the plants were also investigated, and it was found that positive variations in the anatomical structure of the median portion of the main stems and blades of mature foliage leaves were detected in the stressed and SA-treated plants. In conclusion, SA treatment increased the salt stress tolerance of cowpea plants by improving the morphological and physiological attributes of the plants.

## 1. Introduction

The cowpea (*Vigna ungiculata* L.) is legume crop grown for its green pods or dry seeds, which provide protein, vitamins, and minerals. It is also used for other purposes such as fodder and as a green manure crop [1]. It is an important source of food for both humans and animal. Its dry seeds provide an equiponderant source of nutrients with numerous health benefits; they have high levels of protein (20.0–39.4%) and a low content of fat (3.1–30.4%) [2]. It plays a paramount role in the human diet, and nutritionists deem the supplementation of legumes through grain-based diets to be one of the best possible options for mitigating problems associated with protein malnutrition. Its area of cultivation is approximately 14 million hectares, producing 4.5 million tons of cowpeas annually in Africa, Asia, and America [3]. The cowpea has the capability of fixing atmospheric nitrogen (N) and grows well in infertile soils [4]. Hence, it could grow better in arid and semi-arid areas and under stress conditions than other crops.

Salt stress is considered one of the most widespread abiotic stresses and severely hampers crop production, especially in arid and semi-arid areas [5]. Approximately 33% of irrigated croplands are affected by salinity levels to varying degrees, and this may surpass 50% by 2050 [6]. Generally, the exposure of plants to high levels of salt can prevent growth and retard developmental processes in many ways, such as osmotic imbalance, cytotoxicity induced by excrescent Na^+^ and Cl^-^, and nutritional inconsistency [7,8]. At a later stage of development, plants exposed to salinity experience increased oxidative stress because of the production of supernumerary amounts of reactive oxygen species (ROS). This results in oxidative injuries to various cellular macromolecules, such as lipids, proteins, and nucleic acids, which eventually deactivate numerous important cellular processes in plants [1,9]. All the major processes, such as photosynthesis, and the energy of plants are also affected by salinity stress. Salinity reduced the ability of plants to absorb water, leading to growth reduction, as well as to impaired metabolic processes similar to those caused by water stress [10,11] and heat stress [12]. The negative effect of salinity stress on growth, physiological aspects, and productivity has been observed in different plant species, such as the faba bean (*Vicia faba*) [13,14], common bean (*Phaseolus vulgaris*) [15,16], wheat (*Triticum aestivum*) [17], basil (*Ocimum basilicum*) [18], and lupine (Lupinus termis) [19].

The use of photoprotectants for enhancing growth and increasing the productivity of many plant species under abiotic stresses is highly recommended. Among them, salicylic acid (SA) plays an important role in defending plants against both biotic and abiotic stress conditions [20]. Exogenously applied SA has been shown to influence a wide range of plant processes, including stomatal closure, seed germination, fruit yield ion uptake, cell membrane permeability, and photosynthetic rate, including pigment content and growth rate [20,21,22,23]. It also has enhanced the activities of antioxidant enzymes and proline (Pro) content [24]. Although the beneficial effects of SA in terms of salinity have been studied in several crops, such as peppers (*Capsicum annuum*) [25], strawberries (*Fragaria* × *ananassa*) [26], common beans (*P. vulgaris*) [27], cotton (*Gossypium hirsutum*) [28], and rice (*Oryza sativa*) [29], there are few studies on the effects of SA on the anatomical characteristics of cowpea plants under salt stress.

Foliar application of SA resulted in thicker leaflets of beans because of an increase in the thickness of both the midvein and lamina. The thicker lamina may be attributed to an increase in the thickness of both the palisade and spongy tissues, as well as the midvein bundle [30]. Similar results were reported by [31], who found that under drought stress SA enhanced the faba bean’s stem diameter, number of vessels per bundle, leaf lamina thickness, and average diameter of the xylem vessel. They noted that the exogenous application of SA led to minimizing the harmful effects of drought stress and improved the drought tolerance of faba beans.

Foliar application of SA increased the diameter thickness of the stem wall and the hollow pith in lupine plants. The increase in stem wall thickness may be attributed to an increase in the thickness of the epidermis, cortex, fiber strands, phloem, and xylem tissues, as well as the thickness of the parenchymatous area of the pith and vessel diameter. The thickness of the midvein and leaflet lamina were the result of an increase in the thickness of the palisade and spongy tissues, as well as an increase in the size of the midvein bundle [32]. SA of up to 10 mg/L. led to an increase in the thickness of the stem cortex, stem vascular bundles, and root cortex, and an amount of 20 mg/L. decreased these traits compared with the control [33].

The present investigation aimed to study the influence of the foliar spraying of SA on the vegetative growth of cowpeas grown under conditions of salinity. Physiological parameters, as well as the stem and leaf anatomy, were evaluated in the current study.

## 2. Materials and Methods

Seeds of the cowpea (*Vigna unguiculata* L. cv. Dokki 331) were obtained from the Vegetable Production Research Department, Agricultural Research Center (ARC), Giza, Egypt. SA was imported from the Electro Scientific Company, Portland, OR, USA.

### 2.1. Experimental Design

Seeds were grown in pots with a diameter of 25 cm, which were filled with 7 kg of a loamy mixture with clean sand soil at a ratio 1:1 *w*/*w*. Each pot was given nitrogen (N) in 2 g of ammonium sulfate (20.5% N), phosphorus (P) in 1 g of calcium superphosphate (15.4% P_2_O_5_), and potassium (K) in 0.5 g of potassium sulfate (48% K_2_O). Pots were arranged in a randomized complete block design with three replicates. Each replicate included 45 pots, or 5 pots for each treatment. The experiment had 9 treatments as follows:

1. Control; plants were irrigated with tap water.
2. 1000 ppm NaCl + 1000 ppm CaCl_2_ (2000 ppm salinity).
3. 2000 ppm NaCl + 2000 ppm CaCl_2_ (4000 ppm salinity).
4. 3000 ppm NaCl + 3000 ppm CaCl_2_ (6000 ppm salinity).
5. 4000 ppm NaCl + 4000 ppm CaCl_2_ (8000 ppm salinity).
6. 2000 ppm salinity + 100 ppm SA.
7. 4000 ppm salinity + 100 ppm SA.
8. 6000 ppm salinity + 100 ppm SA.
9. 8000 ppm salinity + 100 ppm SA.

Each week for 3 weeks, 500 mL/pot of the different salt concentrations was applied and tap water was added for 1 week during the experiment for leaching.

The first and second applications of 100 ppm of SA were made after 6 and 8 weeks, respectively, from sowing. The volume of sprayed solution per pot was approximately 10 and 18 mL, respectively, using Tween 20 (0.05%) as a surfactant.

### 2.2. Measurement of Vegetative Growth Characters

A random sample of 10 plants from each treatment (two plants from every pot) was assigned for consideration. Vegetative attributes were registered 10 weeks after sowing when the plants were in full bloom. The following characters were studied: Plant height (cm), number of compound leaves per plant, average number of internodes per plant, and fresh weights of leaves and stems per plant in grams.

### 2.3. Yield and Its Components Measurement

A random sample of 12 plants (four plants from each replicate) were randomly taken at 120 days from sowing to determine the following data:

1. Average no. of pods/plant;
2. Average no. of seeds/pod;
3. Weight of 100 seeds (g);
4. Weight of pods (g)/plant.

### 2.4. Determination of Photosynthetic Pigments

Chlorophyll a (Ch_a_), chlorophyll b (Ch_b_), and carotenoids (Car) contents were extracted from upper fresh leaves at the age of 75 days by dimethyl formamide and the contents were estimated according to the Moran [34] developed method.

### 2.5. Estimation of Free Proline Content

The content of free proline was estimated in the fresh leaves as described by Bates et al. [35], using a Spectranic 2000 spectrophotometer (Bausch and Lomb; Irvine, CA, USA). The absorbance was measured at 520 nm.

### 2.6. Determination of N, P, and K Content

Samples from dried leaves were taken after 75 days from sowing of the second growing season to estimate N, P, and K as described by A.O.A.C. [36].

### 2.7. Anatomical Studies

Tested material included main stem and lamina of the terminal leaflet from the developed compound leaf on the median portion of the main stem were taken throughout the second growing season of 2020 at the age of 75 days from the sowing date.

Anatomical characters in cowpea stem:

- Thickness of cortex (µm.);
- Thickness of fiber strands (µm.);
- Thickness of phloem tissue (µm.);
- Thickness of xylem tissue (µm.);
- Mean diameter of vessel (µm.);
- Diameter of the pith (µm.).

Anatomical characters in cowpea leaf:

- Midvein thickness (µm.);
- Lamina thickness (µm.);
- Palisade tissue thickness (µm.);
- Spongy tissue thickness (µm.);
- Length of midvein bundle (µm.);
- Width midvein bundle (µm.);
- No. of xylem rows/midvein bundle;
- Vessel diameter (µm.).

(Each value means 5 sections, 5 readings per each).

The execution of microtechnique was carried out according to the method described by Nassar and El-Sahhar [37].

### 2.8. Statistical Analysis

We used one-way ANOVA to compare the average of the studied traits. The Duncan multiple range test as post-hoc analysis was used to show the differences among the treatments. The statistical package “SPSS for Windows ver. 25” was used to analyze the data.

## 3. Results

### 3.1. Vegetative Growth Characters

**Figure 1:**
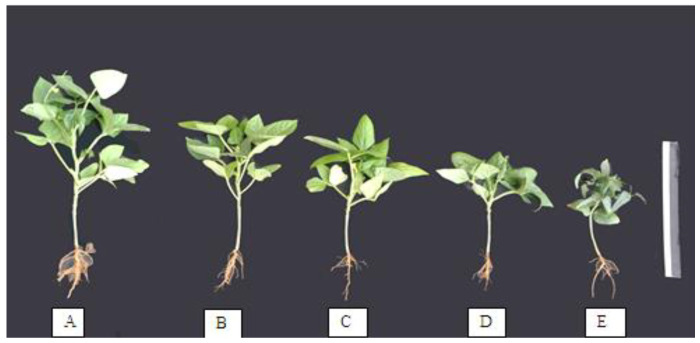
Habit of cowpea plant at blooming stage (the age of 90 days) as affected by salinity stress. (**A**) Untreated plant (control), (**B**) plant treated with 2000 ppm salinity, (**C**) plant treated with 4000 ppm salinity, (**D**) plant treated with 6000 ppm salinity, and (**E**) plant treated with 8000 ppm salinity.

**Figure 2:**
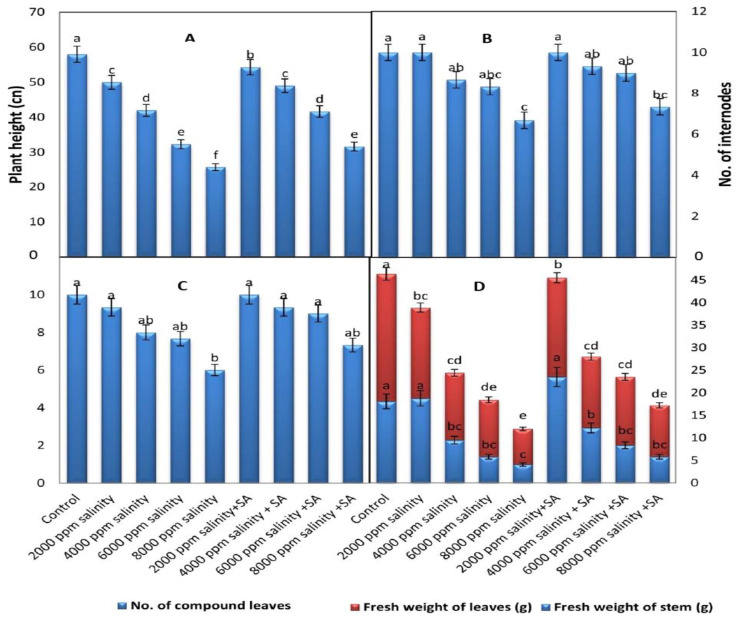
Vegetative growth characters of cowpea as affected by different levels of saline water and foliar application with salicylic acid (SA) at concentration of 100 ppm (average of the two seasons 2019 and 2020 combined). (**A**) Plant height, (**B**) no. of internodes, (**C**) no. of compound leaves, and (**D**) fresh weight of leaves and stem. Data are means of five replicates (*n* = 5), and for each parameter, the mean values ± SD followed by a different letter are significantly different according to Tukey’s HSD range test at *p* ≤ 0.05.

Figure 1 and Figure 2 show that the plant height and fresh weight of shoots were significantly decreased with an increase in salinity levels, and that the number of compound leaves per plant and number of internodes per plant were non-significant compared with control plants.

**Figure 3:**
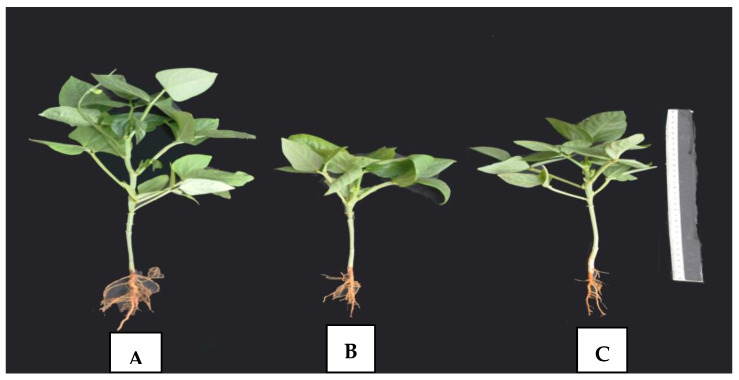
Habit of mature plants, at blooming stage, of cowpea plants as affected by SA and salinity stress. (**A**) Untreated plant (control), (**B**) plant grown under 6000 ppm of salinity stress, and (**C**) plant grown under 6000 ppm salinity stress and sprayed with 100 ppm of SA.

It can be observed in Figure 2 and Figure 3 that the interaction between salinity stress levels and the exogenous application of SA at 100 ppm significantly increased some studied growth characters, such as plant height, number of internodes per plant, number of compound leaves per plant, and fresh weight of shoots. It is clear from Figure 3B,C that the application of SA at 100 ppm had the ability to overcome the deleterious impact of salt stress on cowpea plants, particularly those grown under 6000 ppm of salinity stress.

### 3.2. Physiological Aspects

#### 3.2.1. Photosynthetic Pigments

**Figure 4:**
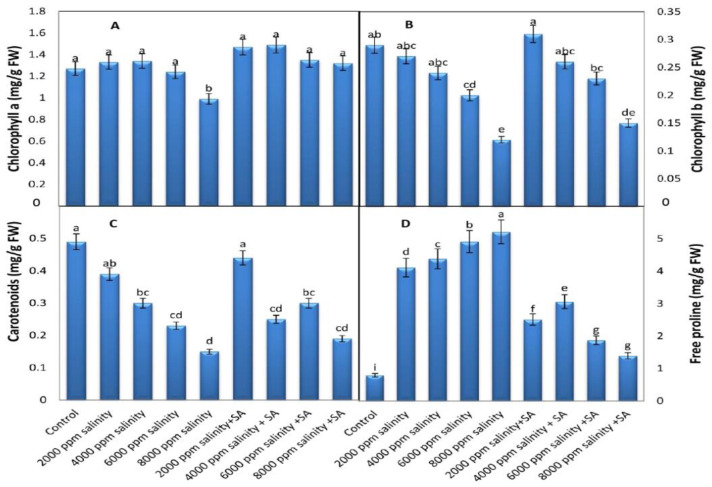
Impact of foliar application with SA on photosynthetic pigments and concentrations of free Pro in cowpea leaves, at full blooming stage, grown under various levels of salinity (average of the two growing seasons, 2019 and 2020 combined). (**A**) Ch_a_, (**B**) Ch_b_, (**C**) Car, and (**D**) Proline. Data are means of five replicates (*n* = 5), and for each parameter, the mean values ± SD followed by a different letter are significantly different according to Tukey’s HSD range test at *p ≤* 0.05.

Figure 4 shows that the salinity significantly decreased the concentration of photosynthetic pigments except for Ch_a_ and Ch_b_ at two low concentrations (2000 and 4000 ppm), where the rate of reduction depended on the concentration. Foliar SA applications at 100 ppm attenuated the harmful effect of salinity on photosynthetic pigments.

#### 3.2.2. Proline Content

There was a significantly increased amount of Pro content in the leaves of the cowpea plants with increased salinity levels, and the rate of increase depended on the concentration of salinity up to 8000 ppm (Figure 4D). Foliar application of SA at 100 ppm decreased the free Pro concentration in the salinized plants.

#### 3.2.3. N, P, and K Concentrations

**Figure 5:**
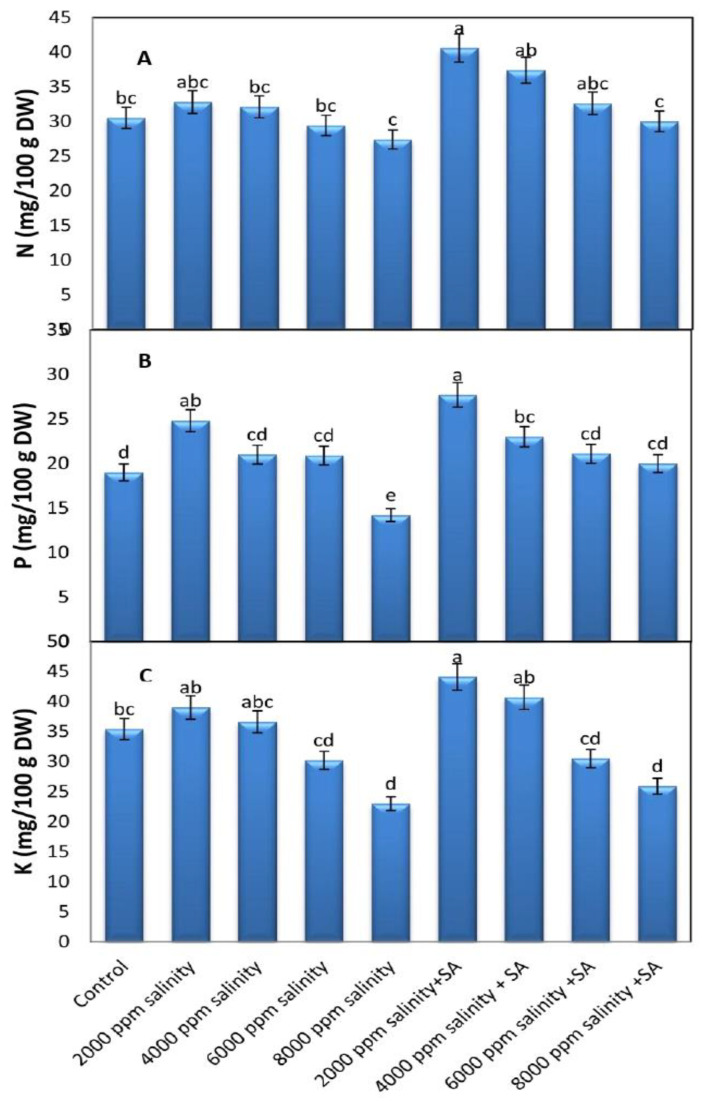
Mean values of N, P, and K in dry leaves of cowpea at full blooming stage, grown under different levels of salinity stress and foliar application with SA (average of the two seasons, 2019 and 2020 combined). (**A**) N content, (**B**) P content, and (**C**) K content. Data are means of five replicates (*n* = 5), and for each parameter, the mean values ± SD followed by a different letter are significantly different according to Tukey’s HSD range test at *p* ≤ 0.05.

It is clear from Figure 5 that salinity at 2000 and 4000 ppm increased the concentrations of N, P, and K in the dried leaves of cowpea plants. Salinity concentrations at 6000 and 8000 decreased these concentrations significantly. The highest levels of N, P, and K were recorded in plants irrigated with 2000 ppm of salinity and sprayed with 100 ppm of SA; they had percentages of 33.11%, 45.7%, and 42.5%, respectively, more than the control plants.

### 3.3. Yield and Its Components

**Figure 6:**
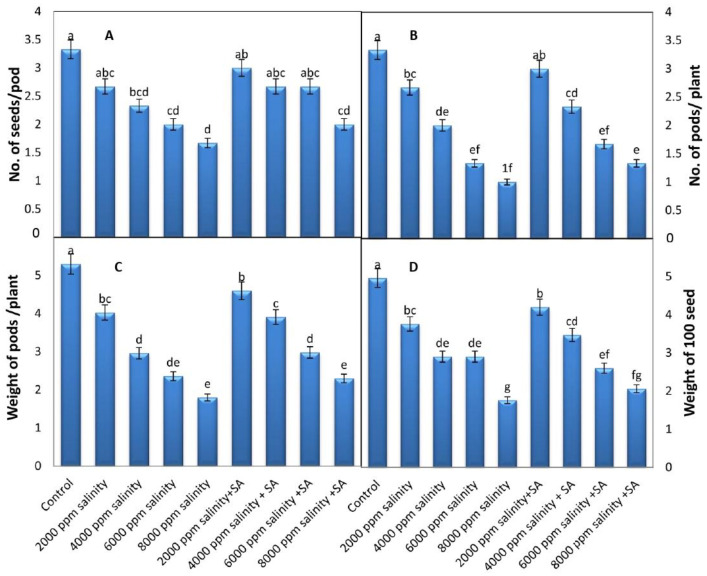
Yield characters of cowpea as affected by different levels of saline water and foliar application with SA at a concentration of 100 ppm (average of the two seasons 2019 and 2020 combined). (**A**) No. of seeds/pod, (**B**) no. of pods/plant, (**C**) weight of pods (g)/plant, and (**D**) weight of 100 seeds. Data are means of five replicates (*n* = 5), and for each parameter, the mean values ± SD followed by a different letter are significantly different according to Tukey’s HSD range test at *p ≤* 0.05.

Results from Figure 6 show that the number of seeds per pod, number of pods per plant, weight of pods per plant, and weight of 100 seeds were significantly decreased in cowpeas that experienced an increase in salinity levels. The highest values of the yield components appeared in the control plants, whereas the lowest values were recorded at a salinity level of 8000 ppm. The interaction between salinity stress (at 6000 ppm) and the exogenous application of SA at 100 ppm, led to a significantly increased number of seeds per pod, number of pods per plant, and weight of pods per plant; the percentages reached 33.5%, 25.6%, and 26.3%, respectively, over those treated with 6000 ppm of salinized water.

### 3.4. Anatomical Studies

#### 3.4.1. Stem Anatomy

**Figure 7:**
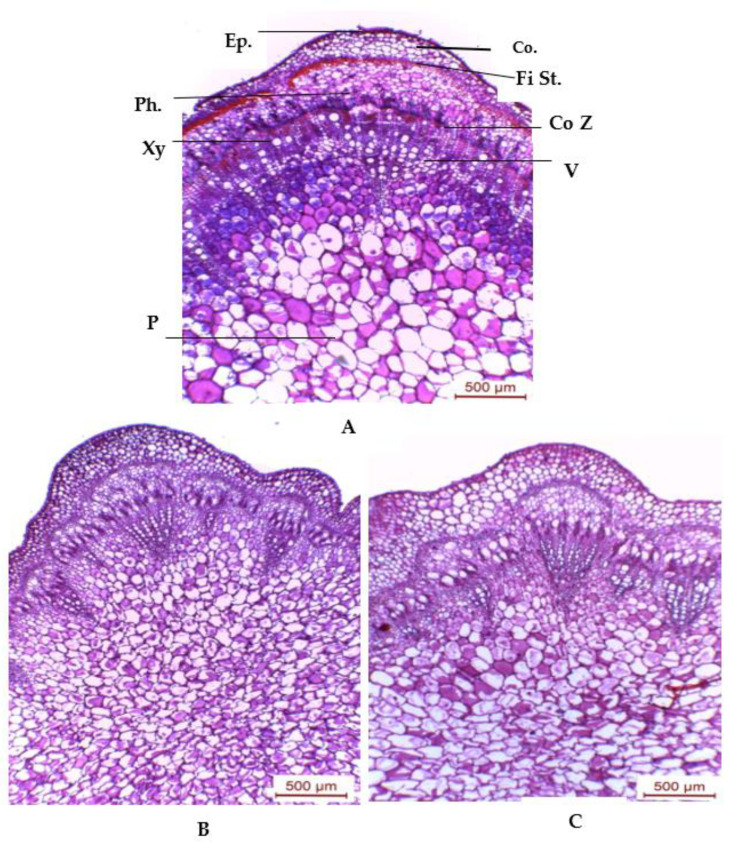
Cross sections through median portion of the main stem of cowpea plant, aged 75 days, as affected by foliar spray with SA. (**A**) Untreated plant (control), (**B**) plant grown under salinity stress of 6000 ppm, and (**C**) plant grown under 6000 ppm salinity stress and sprayed with 100 ppm of SA. Abbreviations: Co Z = cambium zone; Co. = cortex; Ep. = epidermis; Fi St. = fiber strands; Ph. = phloem; P = pith; V = vessel; and Xy = xylem.

Table 1 and Figure 7 show that salinity at 6000 ppm reduced the diameter of the stems, and the thickness of the cortexes, fiber strands, phloem tissues, xylem tissues, mean diameters of the vessels, and piths by 18.8%, 23.5%, 27.0%, 19.4%, 49.5%, 60.2%, and 11.1%, respectively, when compared with the controls.

#### 3.4.2. Leaf Anatomy

**Figure 8:**
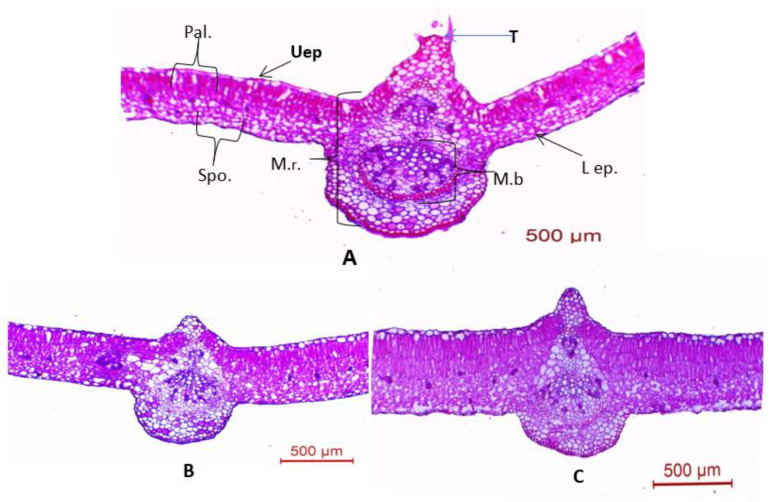
Microphotographs of cross sections through the blade of the terminal leaflet of the compound leaf developed on the median portion of the main stem of cowpea plant, aged 75 days as affected by SA and salinity stress. (**A**) Untreated plant (control), (**B**) plant grown under salinity stress of 6000 ppm, and (**C**) from plant grown under 6000 ppm of salinity stress and sprayed with 100 ppm of SA. Abbreviations: Uep = upper epidermis; L ep., = lower epidermis; Pal. = palisade tissue; Spo. = spongy tissue; M.b = midvein bundle; M.r. = midvein region; and T = trichome.

Data in Table 2 and photomicrographs in Figure 8 indicate that salinity at the level of 6000 ppm decreased the thickness of the midvein, midvein bundle length and width, number of xylem rows per midvein bundle, and vessel diameter by 28.0%, 17.7%, 16.6%, 28.8%, and 14.7%, respectively, when compared with the controls.

Moreover, salinity stress at the level of 6000 ppm increased the thickness of the lamina by 66.8 % over the control. It is worth noting that the foliar application of 100 ppm of SA on cowpea plants grown under a salinity stress of 6000 ppm enhanced the anatomical features of leaves owing to an increase in their thickness in salinized plants. This treatment offset the decreases that occurred in all the included tissues of the leaf blades so that their mean values nearly reached those of the controls. It could be declared that SA had the capability of averting the deleterious impact of salinity stress on the anatomical structure of the cowpea plant leaf. That treatment decreased the effect of salt stress on the thickness of the midvein by 21.6% compared with the controls. The lamina thickness, palisade tissue thickness, and spongy tissue thickness were increased by 43.6%, 28.0%, and 18.9%, respectively, over the controls. The midvein bundles (length and width), as well as the number of xylem rows per midvein bundle and vessel diameters, were decreased by 11.0%, 10.53%, 18.6%, and 8.6%, respectively, compared with the controls. It is noteworthy that the values of all tissues in leaves of plants exposure to salinity that were sprayed with SA were definitely higher than the values for salinized plants. The thickness of the midvein, length, and width of the midvein bundle, number of xylem rows per midvein bundle, and vessel diameter were increased by 8.98%, 7.3%, 7.3%, 14.2%, and 7.0%, respectively, over the controls. The lamina was increased by 13.2% and 24.1% compared with plants treated with 6000 ppm of salinized water.

## 4. Discussion

Salt stress is considered one of the major problems in the agricultural world. It has caused severe reductions in the yield and quality of stressed plants. SA is a plant hormone that regulates plant development and diverse physiological and biochemical mechanisms in plants, especially under stress conditions. Our results show that SA mitigates the negative effects of salt stress on the growth, physiological and anatomical characteristics, and productivity of cowpea plants.

The decrease of plant growth under salt stress may be due to the reduction in cell division and elongation [38]; there is also a destructive impact of NaCl on different physiological pathways and molecular changes through impaired photosynthesis, nutrient imbalances, stomatal impedance to water flow, alterations in the ultrastructure of chloroplasts and mitochondria (which affect the natural metabolism), and hormonal imbalances [39,40]. Earlier studies [25,26,28,41] showed that SA could reduce salt damage in various plants. The exogenous application of SA is expected to control stomatal opening under stress, and this decreases transpiration and water loss, sustains turgor, and controls plant growth and productivity under stress conditions [33,42].

High levels of salinity decreased photosynthetic pigments and this pernicious influence of salinity may be due to increments of the chlorophyllase enzyme, deterioration of the photosynthetic rate, and CO_2_ assimilation and stomatal motion [43]. In addition, there is a decline in chlorophyll biosynthesis intermediation concentrations [44]. During salinity stress, the increased production of oxygen radicals in cells causes oxidation and, hence, the degradation of photosynthetic pigments [45]. SA reduces the harmful effect of salinity on chlorophyll by decreasing the ROS and elevating the antioxidant systems. This enhances plant growth by activating cell division and elongation and perhaps by inhibiting chlorophyll oxidase enzymes, subsequently preventing chlorophyll breakdown and enhancing photosynthesis [46,47].

Salinity stress increases the Pro content and this increases an important mechanism to reduce the harmful effect of salt stress and improve plant growth [48]. Earlier reports [49,50,51] cleared the accumulation of Pro under salinity. The foliar application of SA under salinity may reduce the devastating effect of salt stress on plant growth and ameliorate Pro accumulation [52].

In this research, the results show that increased salinity of up to 8000 ppm decreased concentrations of N, P, and K (Figure 5). This may be due to an ionic imbalance [26] resulting from a disorder in the integrity of the plasma membranes of cells [53] in addition to a converse relationship between Na and other elements [54]. SA influences numerous important physiological functions in plants, such as increasing the uptake of nutrients and decreasing Na^+^ concentrations under salt stress [22,23]. Similarly, [26] researchers have reported that increasing the concentration of SA up to 90 ppm mitigates the negative effect of salinity on N, P, and K concentrations and decreases the Na^+^ content of strawberry leaves under high levels of NaCl (40 mM). It was reported that the foliar supplementation of SA at 0, 1.0, 1.5, and 2.0 mM reduced the Na uptake of plants and was efficient in enhancing nutrient uptake by cotton seedlings grown under 150 mM NaCl [28]. Moreover, SA at 1.0 mmol/L plus NaCl treatment significantly improved nutrient accumulation and also decreased Na^+^ and Cl^−^ accumulations compared with NaCl treatment [29].

A decrease in yield under salinity conditions was probably due to the reduction in photoassimilation assembly, as well as photoassimilation mobilization, which induced a decrease in the harvest index [55]. The reductions of yield may be due to the reverse impact of salt stress on growth characteristics and physiological operations such as water uptake, photosynthesis, flowering, and pod formation, which led to a decreased yield [52]. Nevertheless, in the present study, SA application ameliorated salinity tolerance in cowpeas and enhanced the plants’ yields. The function of SA in yields is probably due to its fundamental role in stress tolerance. It decreases oxidative damage and increases plant growth and yields under salinity stress [56], and it also improves nutrient uptake and increases the levels of chlorophyll and carotenoid pigments, modulates the activities of several enzymes, and conserves the integrity of cell membranes [22,23]. It also plays a role in balancing plant hormones [57].

Anatomical characters were decreased by salinity in cowpea plants. Research by [58] found that salinity decreased the thickness of the upper epidermal layer, cortex layers, hypodermal layer, and pith area. It was noticed that spraying 100 ppm of SA on cowpea plants exposed to 6000 ppm of salinity stress enhanced anatomical features of the main stem. A study by [59] found that drought stress at 40% or 20% reduced the upper and lower epidermis, midvein thickness of the leaflet blade (in length and width), number of xylem rows per midvein bundle, and vessel diameter of cowpea plants. Salinity at 6000 ppm decreased the thickness of both the lamina and midvein of the mature foliage leaf of the basil plant [18].

SA had the capability of avoiding the deleterious impact of salt stress on the anatomical structure of the cowpea plant. In the present study, the foliar application of 100 ppm of SA on cowpea plants exposed to 6000 ppm of salinity stress enhanced the anatomical features of leaves. These results are in accordance with those observed by [30].

## 5. Conclusions

The results of this study revealed that the exogenous spraying of SA might alleviate the inhibitory impact of salinity stress on the growth, physiological and anatomical features, and the productivity of cowpea plants.

## Figures and Tables

**Table 1:** Measurements (µm) of some anatomical aspects in cross-sections through the median portion of the main stem of cowpea plant, aged 75 days, grown under salt stress, and influenced by spraying with salicylic acid (SA).

	Treatments					
Histological Characters	Control	6000 ppm Salinity	±Percentage to Control	6000 ppm Salinity + 100 ppm SA	±Percentage to Control	±Percentage to 6000 ppm Salinity
Thickness of cortex	5820.6	4723.8	−18.8	5104.4	−12.3	+8.0
Thickness of fiber strands	5820.6	267.7	−23.5	302.8	−13.5	+13.1
Thickness of phloem tissue	41.8	53.1	−27.0	81.1	+94.0	+52.7
Thickness of xylem tissue	217.2	175.0	−19.4	193.8	−10.7	+10.7
Mean diameter of vessel	700.1	353.1	−49.5	436.7	−37.6	+23.6
Diameter of the pith	66.1	26.3	−60.2	42.5	−35.7	+61.5

**Table 2:** Counts and measurements in micro-meters (µm) of certain histological characters in transverse sections through the blade of the terminal leaflet of the compound leaf developed on the median portion of the main stem of cowpea plant, aged 75 days, grown under salt stress, and influenced by spraying with SA.

	Treatments					
Histological Characters	Control	6000 ppm Salinity	±Percentage to Control	6000 ppm Salinity + 100 ppm SA	±Percentage to Control	±Percentage to 6000 ppm Salinity
Midvein thickness	1315.2	945.8	−28.0	1030.8	−21.6	+8.98
Lamina thickness	306.2	500.0	+66.8	439.9	+43.6	−12.02
Palisade tissue thickness	203.7	302.2	+30.1	260.8	+28.0	−13.6
Spongy tissue thickness	102.1	198.8	+94.7	121.4	+18.9	−38.9
Length of midvein bundle	640.5	530.5	−17.7	569.7	−11.0	+7.3
Width midvein bundle	481.1	400.9	−16.6	430.4	−10.53	+7.3
No. of xylem rows/midvein bundle	5.9	4.2	−28.8	4.8	−18.6	+14.2
Vessel diameter	29.9	25.5	−14.7	27.3	−8.6	+7.0
